# Ileum Gene Expression in Response to Acute Systemic Inflammation in Mice Chronically Fed Ethanol: Beneficial Effects of Elevated Tissue n-3 PUFAs

**DOI:** 10.3390/ijms22041582

**Published:** 2021-02-04

**Authors:** Josiah E. Hardesty, Jeffrey B. Warner, Ying L. Song, Eric C. Rouchka, Craig J. McClain, Dennis R. Warner, Irina A. Kirpich

**Affiliations:** 1 Division of Gastroenterology, Hepatology, and Nutrition, Department of Medicine, University of Louisville, Louisville, KY 40202, USA; josiah.hardesty@louisville.edu (J.E.H.); jeffrey.warner.1@louisville.edu (J.B.W.); ying.song@louisville.edu (Y.L.S.); craig.mcclain@louisville.edu (C.J.M.); dennis.warner@louisville.edu (D.R.W.); 2 Department of Pharmacology and Toxicology, School of Medicine, University of Louisville, Louisville, KY 40202, USA; 3 Department of Computer Science and Engineering, Speed School of Engineering, University of Louisville, Louisville, KY 40292, USA; eric.rouchka@louisville.edu; 4 University of Louisville Alcohol Center, School of Medicine, University of Louisville, Louisville, KY 40292, USA; 5 University of Louisville Hepatology and Toxicology Center, School of Medicine, University of Louisville, Louisville, KY 40292, USA; 6 Robley Rex Veterans Medical Center, Louisville, KY 40206, USA

**Keywords:** alcohol, acute systemic inflammation, polyunsaturated fatty acids, intestine, transcriptome

## Abstract

Chronic alcohol consumption leads to disturbances in intestinal function which can be exacerbated by inflammation and modulated by different factors, e.g., polyunsaturated fatty acids (PUFAs). The mechanisms underlying these alterations are not well understood. In this study, RNA-seq analysis was performed on ileum tissue from WT and *fat-1* transgenic mice (which have elevated endogenous n-3 PUFAs). Mice were chronically fed ethanol (EtOH) and challenged with a single lipopolysaccharide (LPS) dose to induce acute systemic inflammation. Both WT and *fat-1* mice exhibited significant ileum transcriptome changes following EtOH + LPS treatment. Compared to WT, *fat-1* mice had upregulated expression of genes associated with cell cycle and xenobiotic metabolism, while the expression of pro-inflammatory cytokines and pro-fibrotic genes was decreased. In response to EtOH + LPS, *fat-1* mice had an increased expression of genes related to antibacterial B cells (APRIL and IgA), as well as an elevation in markers of pro-restorative macrophages and γδ T cells that was not observed in WT mice. Our study significantly expands the knowledge of regulatory mechanisms underlying intestinal alterations due to EtOH consumption and inflammation and identifies the beneficial transcriptional effects of n-3 PUFAs, which may serve as a viable nutritional intervention for intestinal damage resulting from excessive alcohol consumption.

## 1. Introduction

The intestinal epithelium serves several important functions including maintenance of the gut barrier and segregation of host tissue from bacteria and bacterial products [1]. To this end, the intestinal epithelium undergoes continuous breakdown and restoration, a process which may be beneficially or detrimentally affected by external or internal factors, such as dietary fatty acids and inflammation [1,2]. For example, n-3 polyunsaturated fatty acid (PUFA) supplementation has beneficial effects with regard to intestinal health and function and the gut microbiome in humans [2,3]. In animals, n-3 PUFA supplementation improved intestinal tight junction integrity and reduced inflammation in a colitis model [4]. Similarly, transgenic mice that endogenously produce n-3 PUFAs from n6 PUFAs (*fat-*1 mice [5]) have reduced intestinal permeability when fed a hypercaloric diet [6] and are also protected from colitis [7]. Whereas n-3 PUFAs exert positive effects on intestinal health and function, dietary n-6 PUFAs (specifically in combination with chronic ethanol [EtOH] consumption) can disrupt intestinal barrier integrity, alter the gut microbiota [8,9,10], and lead to intestinal inflammation and abnormal gut barrier defense [11]. A compromised gut barrier allows bacteria and bacterial products such as lipopolysaccharide (LPS) from gram-negative bacteria to translocate to the blood and other organs [12], causing systemic inflammation and further exacerbating gut permeability and intestinal injury [13]. In addition, chronic intestinal inflammation can also promote intestinal fibrosis, leading to additional loss of gut barrier function [14]. We recently reported that *fat-1* mice had attenuated EtOH-induced alterations in intestinal homeostasis [15] that were associated with a markedly plastic transcriptome response to EtOH as well as specific transcriptional signatures [16]. Cell death, inflammation, and tuft cell markers were downregulated in *fat-1* mice in response to EtOH, while defense responses and PPAR signaling were upregulated [16]. Importantly, however, the molecular mechanisms underlying EtOH and EtOH + LPS-mediated intestinal pathology remain poorly understood.

Since EtOH consumption and acute systemic inflammation contribute to gut pathology, we aimed to elucidate the associated transcriptional responses using an unbiased RNA-seq based approach. Previously, we demonstrated improved intestinal health with n-3 PUFA enrichment in *fat-1* transgenic mice (the mice that endogenously convert n6 to n3 PUFAs [15]) and identified the associated ileum transcriptional responses to EtOH [16]. The current study builds upon our previous work and aims to identify n-3 PUFA-regulated ileum transcriptional responses to acute systemic inflammation in mice chronically fed EtOH.

## 2. Results

### 2.1. Chronic EtOH Consumption Followed by LPS Challenge Leads to Global Ileum Gene Changes in Both WT and fat-1 Mice

**Figure 1:**
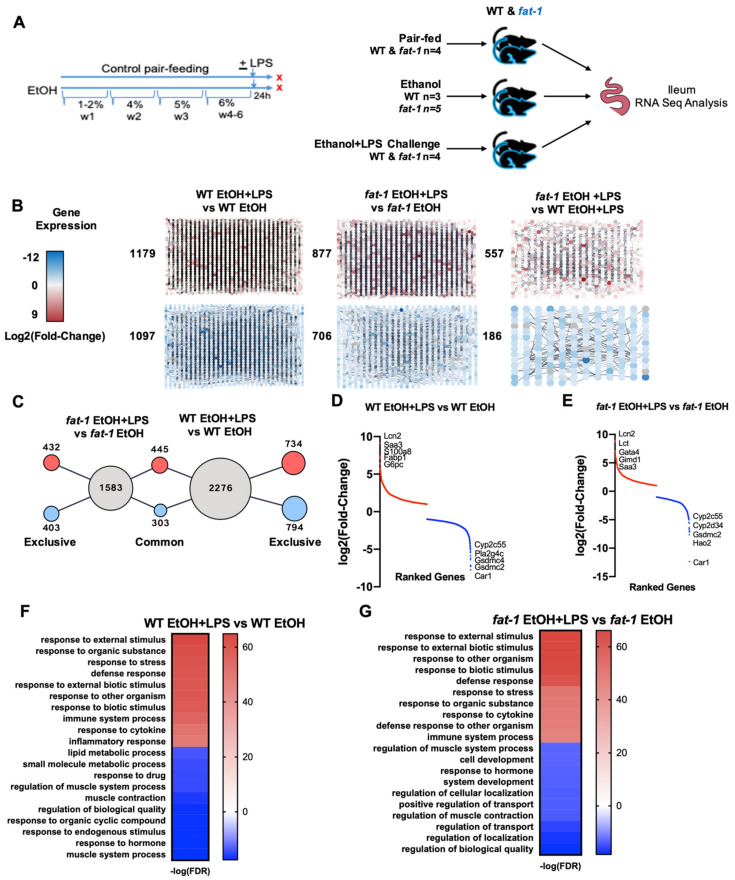
Chronic EtOH consumption followed by LPS challenge leads to global ileal gene changes in both WT and *fat-1* mice. (**A**) WT and *fat-1* mice were either pair-fed (WT *n* = 4, *fat-1 n* = 4), EtOH-fed (WT *n* = 3, *fat-1 n* = 5), or EtOH-fed + a one-time injection of LPS 24 h before sacrifice (WT-EtOH + LPS *n* = 4, fat-1 EtOH + LPS *n* = 4).(**B**) Gross RNA-seq data from WT EtOH + LPS vs. WT EtOH mice, *fat-1* EtOH + LPS vs. *fat-1* EtOH mice, and *fat-1* EtOH + LPS vs. WT EtOH + LPS mice. Nodes in the red gradient were increased for the given comparison and nodes in the blue gradient were decreased for the given comparison, with the total number of genes listed to the side. (**C**) The number of gene expression changes in response to EtOH + LPS, either exclusive or common to genotype. (**D**) Plot of log_2_ (Fold-change ranked genes) for the WT EtOH + LPS vs. WT EtOH comparison (red increased, blue decreased). (**E**) Plot of log_2_ (Fold-change ranked genes) for the *fat-1* EtOH + LPS vs. *fat-1* EtOH comparison (red increased, blue decreased). (**F**) Heatmap of GO processes for the WT EtOH + LPS vs. WT EtOH comparison. (**G**) Heatmap of GO processes for the *fat-1* EtOH + LPS vs. *fat-1* EtOH comparison.

To determine the effect of chronic EtOH consumption and LPS challenge on ileal gene expression, WT and *fat-1* mice were fed an EtOH-containing diet with or without a single LPS challenge followed by ileum RNA-seq analysis (Figure 1A). There were 2276 differentially expressed genes (DEGs) in the ileum between WT EtOH+LPS vs. WT EtOH mice, 1583 DEGs between *fat-1* EtOH + LPS vs. *fat-1* EtOH mice, and 743 DEGs between *fat-1* EtOH + LPS vs. WT EtOH + LPS (Figure 1B). There were 734 genes increased and 794 decreased exclusively in WT mice, and 435 genes increased and 403 decreased exclusively in *fat-1* mice in response to EtOH + LPS vs. EtOH. There were commonly altered genes by LPS (445 genes increased and 303 decreased) in both WT and *fat-1* EtOH-fed mice (Figure 1C). A Venn diagram of the number of significant genes between *fat-1* EtOH + LPS vs. *fat-1* EtOH, WT EtOH + LPS vs. WT EtOH, and *fat-1* EtOH + LPS is included as Figure S1. The most upregulated genes in the WT EtOH + LPS vs. WT EtOH comparison included *Lcn2*, *Saa3*, *S100a8*, *Fabp1*, and *G6pc* (568.8-, 166.6-, 140.1-, 88.3-, and 80.8-fold, respectively), while the downregulated genes included *Cyp2c55*, *Pla2g4c*, *Gsdmc4*, *Gsdmc2*, and *Car1* (-215-, -155.7-, -149.9-, -141.2-, and -84.4-fold, respectively) (Table S1 and Figure 1D). The most upregulated genes in the *fat-1* EtOH+LPS vs. *fat-1* EtOH comparison included *Lcn2*, *Lct*, *Gata4*, *Gimd1*, and *Saa3* (496.8-, 402-, 134-, 123.9-, and 122.2-fold, respectively), while the downregulated genes included *Cyp2c55*, *Cypd34*, *Gsdmc2*, *Hao2*, and *Car1* (-197.9-, -143.9-, -127.1-, -122.3-, and -99.1-fold, respectively) (Table S2 and Figure 1E). Gene Ontology (GO) processes increased in EtOH + LPS vs. EtOH-treated WT mice included response to stress, defense response, and inflammatory processes. In contrast, diminished GO processes included lipid metabolism, muscle contraction, and response to hormones (Figure 1F). GO processes enriched in *fat-1* mice in response to EtOH + LPS vs. EtOH included immune system response and defense responses. Diminished GO processes included transport, development, and muscle contraction (Figure 1G).

### 2.2. Similarity in Transcriptional Responses of Ileum Tissue to EtOH+LPS in WT and fat-1 Mice

**Figure 2:**
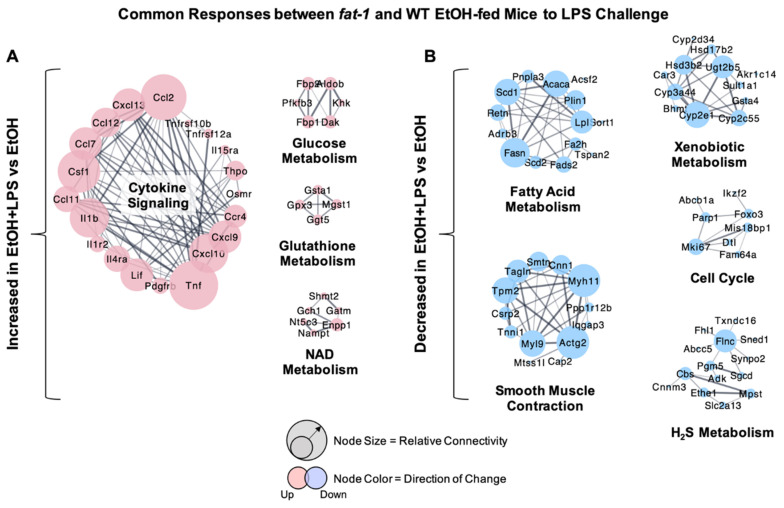
Similarity in transcriptional responses of ileum tissue to EtOH + LPS in WT and *fat-1* mice. (**A**) Cluster analysis of ileum genes increased by EtOH + LPS vs. EtOH in both WT and *fat-1* mice. Node size indicates relative connectivity. (**B**) Cluster analysis of ileum genes decreased by EtOH + LPS vs. EtOH in both WT and *fat-1* mice.

Analysis of gene expression changes between WT and *fat-1* mice revealed a number of similar changes in both. The most upregulated ileum genes in response to EtOH + LPS vs. EtOH in both genotypes included *Lcn2*, *Gata4*, *Gimd1*, *Saa3*, and *Plb1* (496.8-, 134-, 123.9-, 122.2-, and 117.5-fold, respectively), while the genes with the greatest decreases included *Car1*, *Cyp2c55*, *Hao2*, *Cyp2d34*, and *Gsdmc2* (-5168.5-, -197.9-, -143.9-, -127.1-, and -122.3-fold, respectively) (Table S3). Cluster analysis identified gene groups that were similarly affected in EtOH + LPS vs. EtOH-treated WT and *fat-1* mice. The expression of genes involved in cytokine signaling (*Tnf*, *Il1b*, *Csf1*), glucose metabolism (*Aldob*, *Fbp1*, *Fbp2*), glutathione metabolism (*Gpx3*, *Gsta1*, *Ggt5*), and nicotinamide adenine dinucleotide (NAD) metabolism (*Nampt*, *Shmt2*, *Enpp1*) were similarly enriched between genotypes (Figure 2A). Gene clusters with diminished expression included those for fatty acid metabolism (*Fasn*, *Acaca*, *Scd1*), smooth muscle contraction (*Actg2, Myh11*, *Tpm2*), xenobiotic metabolism (*Cyp2e1*, *Ugt2b5*, *Cyp3a44*), cell cycle (*Mki67*, *Foxo3*, *Parp1*), and H_2_S metabolism (*Flnc*, *Cbs*, *Mpst*) (Figure 2B).

### 2.3. Increased Transcriptional Responses to EtOH + LPS Challenge Specific to WT or fat-1 Mice

**Figure 3:**
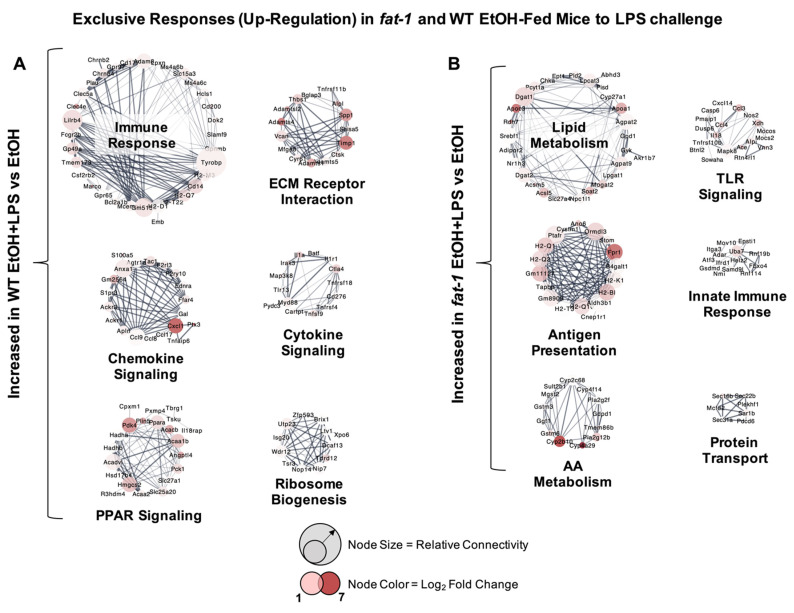
Exclusive transcriptional responses increased due to EtOH + LPS in WT and *fat-1* mice. (**A**) Cluster analysis of the expression of genes increased exclusively in WT mice in response to EtOH + LPS vs. EtOH. (**B**) Cluster analysis of ileum genes increased exclusively in *fat-1* mice in response to EtOH + LPS vs. EtOH. Node size indicates relative connectivity. Node color indicates relative log_2_(Fold-Change) of genes.

There were many transcriptional changes that were unique to each genotype. The most highly upregulated ileum genes exclusive to WT EtOH + LPS vs. WT EtOH-treated mice included *Fabp1*, *S100a8*, *Igkv3-1*, *Ptx3*, and *Fga* (166.6-, 80.7-, 47.5-, 44.6-, and 43.7-fold, respectively) (Table S4). Ileum genes significantly upregulated exclusively in *fat-1* EtOH + LPS vs. *fat-1* EtOH mice included *Lct*, *Ugt2a3*, *Cyp2b10*, *Enpp3*, and *Fpr1*(402-, 91.6-, 64.6-, 53-, and 33.8-fold, respectively) (Table S5). Next, cluster analysis of genotype-exclusive gene sets in WT and *fat-1* mice exposed to EtOH + LPS vs. EtOH was performed. Gene clusters upregulated in EtOH + LPS vs. EtOH-treated WT mice included those for immune response (*Lilrb4*, *Cd14*, *Tyrobp*), chemokine signaling (*Cxcl1, Anxa1*, *Ccl9*), peroxisome proliferator-activated receptor (PPAR) signaling (*Pparα*, *Hmgcs2*, *Acaa1b*), extracellular matrix (ECM) receptor interaction (*Timp1*, *Thbs1*, *Adamts4*), cytokine signaling (*Il1a*, *Il1r1*, *Myd88*), and ribosome biogenesis (*Wdr12*, *Tdrd12*, *Utp23*) (Figure 3A). Gene sets that were exclusively upregulated in EtOH + LPS vs. EtOH-treated *fat-1* mice included those for lipid metabolism (*Nr1h3*, *Apoc3*, *Srebf1*), antigen presentation (*Fpr1*, *H2-Q1*, *H2-Q2*), amino acid (AA) metabolism (*Cyp2b10*, *Cyp4a29*, *Gstm6*), toll-like receptor (TLR) signaling (*Ccl3*, *Ccl4*, *Nos2*), innate immune response (*Uba7*, *Adar*, *Atf3*), and protein transport (*Sec16b*, *Sar1b*, *Sec22b*) (Figure 3B).

### 2.4. Decreased Transcriptional Responses to EtOH+LPS Challenge Specific to WT or fat-1 Mice

**Figure 4:**
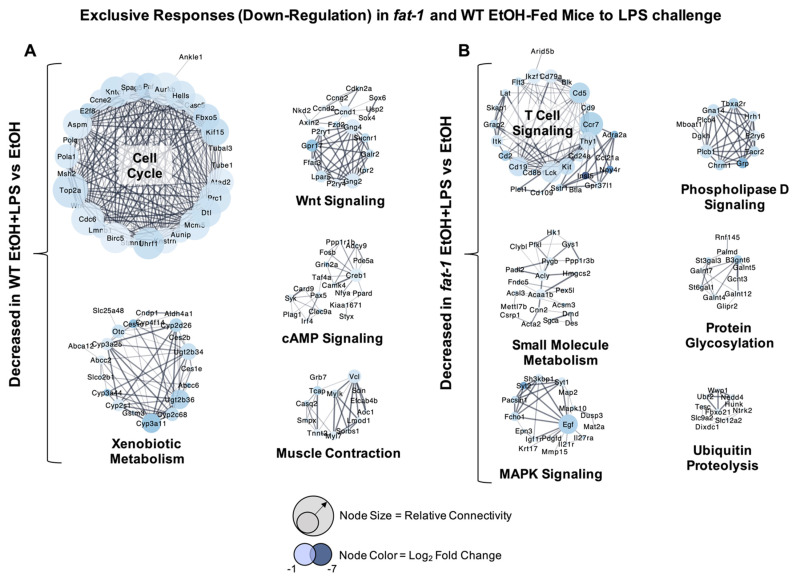
Exclusive transcriptional responses decreased due to EtOH + LPS in WT and *fat-1* mice. (**A**) Cluster analysis of ileum genes decreased exclusively in WT mice in response to EtOH + LPS vs. EtOH. (**B**) Cluster analysis of ileum genes decreased exclusively in *fat-1* mice in response to EtOH + LPS vs. EtOH. Node size indicates relative connectivity. Node color indicates relative log_2_(Fold-Change) of genes.

There were also genes whose expression was decreased by EtOH + LPS vs. EtOH exclusively in WT and *fat-1* mice. The most highly downregulated genes exclusive to WT EtOH + LPS vs. WT EtOH-treated mice included *Mme*, *Igkv2-109*, *Igkv4-53*, *Ighv14-4*, and *Rn7sk* (-33.1-, -31.2-, -24.5-, -19.8-, and -17.8-fold, respectively) (Table S4). Genes that were highly downregulated exclusively in *fat-1* EtOH + LPS vs. *fat-1* EtOH mice included *Nov*, *Grin3a*, *Ighg2c*, *Npy4r*, and *Gm11346* (-86.8-, -45-, -32.8-, -29.2-, and -28.9-fold, respectively) (Table S5). Ileum gene clusters with decreased expression in WT mice in response to EtOH + LPS vs. EtOH included those for cell cycle (*Top2a*, *Aurkb*, *Pola1*), xenobiotic metabolism (*Cyp3a11*, *Cyp2c68*, *Cyp3a44*), Wnt signaling (*Axin2*, *Fzd2*, *Nkd2*), cyclic adenosine monophosphate (cAMP) signaling (*Creb1*, *Pde5a*, *Fosb*), and muscle contraction (*Myl7*, *Mylk*, *Vcl*) (Figure 4A). Gene clusters with decreased expression exclusively in *fat-1* mice in response to EtOH + LPS vs. EtOH included those for T cell signaling (*Thy1*, *Ccr7*, *Cd8b*), small molecule metabolism (*Acly*, *Acaa1b*, *Pygb*), mitogen-activated protein kinase (MAPK) signaling (*Mapk10*, *Igf1r*, *Map2*), phospholipase D signaling (*Grp*, *Plcb1*, *Dhkh*), protein glycosylation (*B3gnt6*, *St3gal3*, *St6gal1*), and ubiquitin proteolysis (*Nedd4*, *Ubr2*, *Wwp1*) (Figure 4B).

### 2.5. Differential Transcriptional Responses between fat-1 EtOH+LPS and WT EtOH + LPS-Exposed Mice

**Figure 5:**
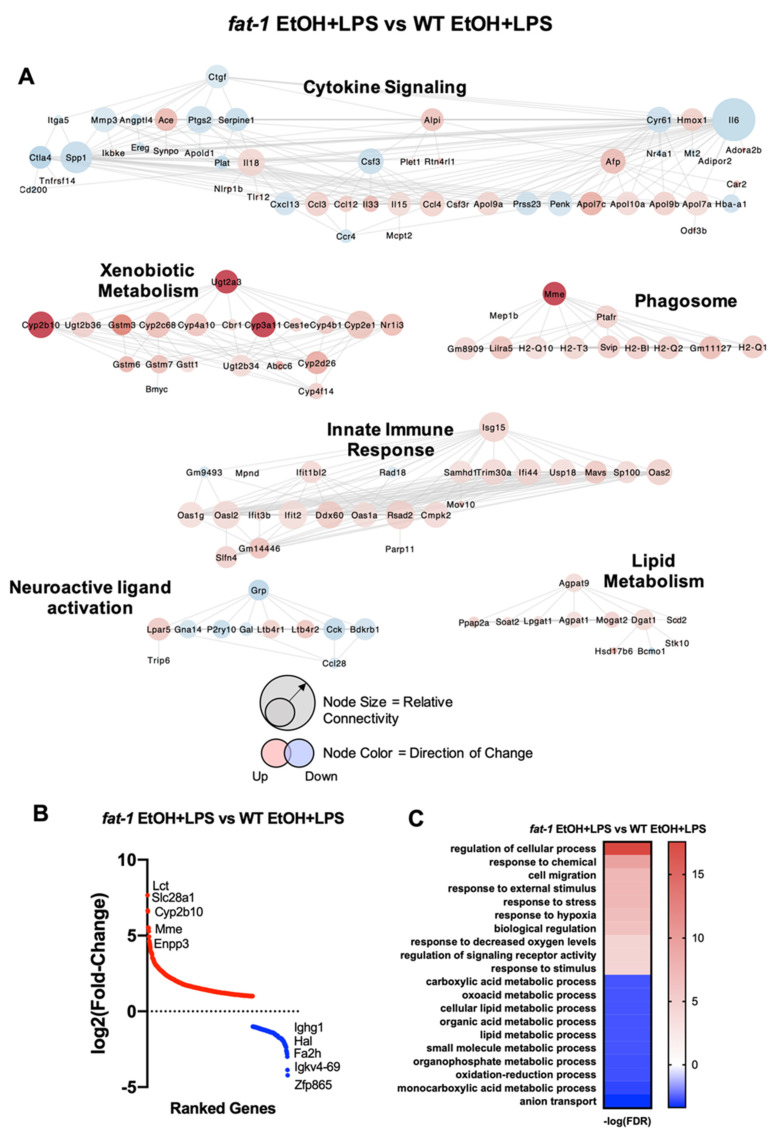
Differential transcriptional responses in *fat-1* EtOH + LPS vs. WT EtOH + LPS-treated mice. (**A**) Cluster analysis of ileum genes differentially expressed between *fat-1* EtOH + LPS vs. WT EtOH + LPS-treated mice. Node size indicates relative connectivity. Node color indicates relative log_2_ (Fold-Change) of genes. (**B**) Plot of log_2_(Fold-change ranked genes) for the *fat-1* EtOH + LPS vs. WT EtOH + LPS comparison (red increased, blue decreased). (**C**) Heatmap of GO processes for the *fat-1* EtOH + LPS vs. WT EtOH + LPS comparison.

Cluster analysis of ileum genes differentially expressed between *fat-1* EtOH + LPS vs. WT EtOH + LPS mice revealed several significant groups (Figure 5A). The cytokine signaling gene cluster was overrepresented for this comparison, which included the following genes: *Il15*, *Il33*, *Serpine1* (increased), *Cxcl13*, *Il6*, and *Itga5* (decreased). Ileum gene clusters enriched in *fat-*1 EtOH + LPS vs. WT EtOH + LPS mice also included genes for xenobiotic metabolism (*Cyp2b10*, *Cyp3a11*, *Ugt2a3*), phagosome (*Mme*, *H2-Q10*, *H2-Q2*), innate immune response (*Oas2*, *Isg15*, *Cmpk2*), and lipid metabolism (*Agpat9*, *Dgat1*, *Soat2*). The neuroactive ligand activation gene cluster had decreased expression of many genes in *fat-1* EtOH + LPS vs. WT EtOH + LPS-treated mice, including *Grp*, *Cck*, and *Gal.* The most upregulated genes in *fat-1* EtOH + LPS vs. WT EtOH + LPS-treated mice included *Lct*, *Slc28a1*, *Cyp2b10*, *Mme*, and *Enpp3* (202.9-, 100.9-, 96.7-, 46.4-, and 45.9-fold, respectively) with the most downregulated genes being *Ighg1*, *Hal*, *Fa2h*, *Igkv4-69*, and *Zfp865* (-18.6-, -14.6-, -8-, -7.1-, and -6.7-fold, respectively) (Table S6) (Figure 5B). GO processes enriched in *fat-1* EtOH + LPS vs. WT EtOH + LPS mice included response to stress, response to hypoxia, and signaling receptor activity, while the diminished processes included anion transport, organophosphate metabolism, and carboxylic acid metabolism (Figure 5C).

### 2.6. Targeted Analysis of Selected Transcriptional Responses Involved in EtOH + LPS-Mediated Alterations of Intestinal Immunity and Intestinal Tissue Integrity

Specific gene sets were investigated to further delineate the effects of chronic EtOH administration in combination with acute inflammation, as well as the role of n-3 PUFA enrichment, on processes related to intestinal immunity and fibrotic intestinal tissue alterations, which are key drivers of intestinal homeostasis and pathology, respectively [1]. To this end, Butyrophilin-like (Btnl) genes, gene markers of intestinal γδ T cells, macrophages, IgA + B cells, and drivers of intestinal tissue fibrosis were evaluated in *fat-1* and WT mice challenged with EtOH + LPS. We also evaluated effects of chronic EtOH exposure alone on specific mediators and pathways involved in intestinal immunity and fibrotic processes by comparing the pair-fed (PF) and EtOH-fed WT and *fat-1* mice; these data are presented in Supplemental Materials Figures S2–S4.

#### 2.6.1. Ileum Gene Expression of Butyrophilin-Like (Btnl) Genes and Markers of γδ T Cells and Pro-Restorative Macrophages Were Enhanced in *fat-1* vs. WT EtOH + LPS-Exposed Mice

**Figure 6:**
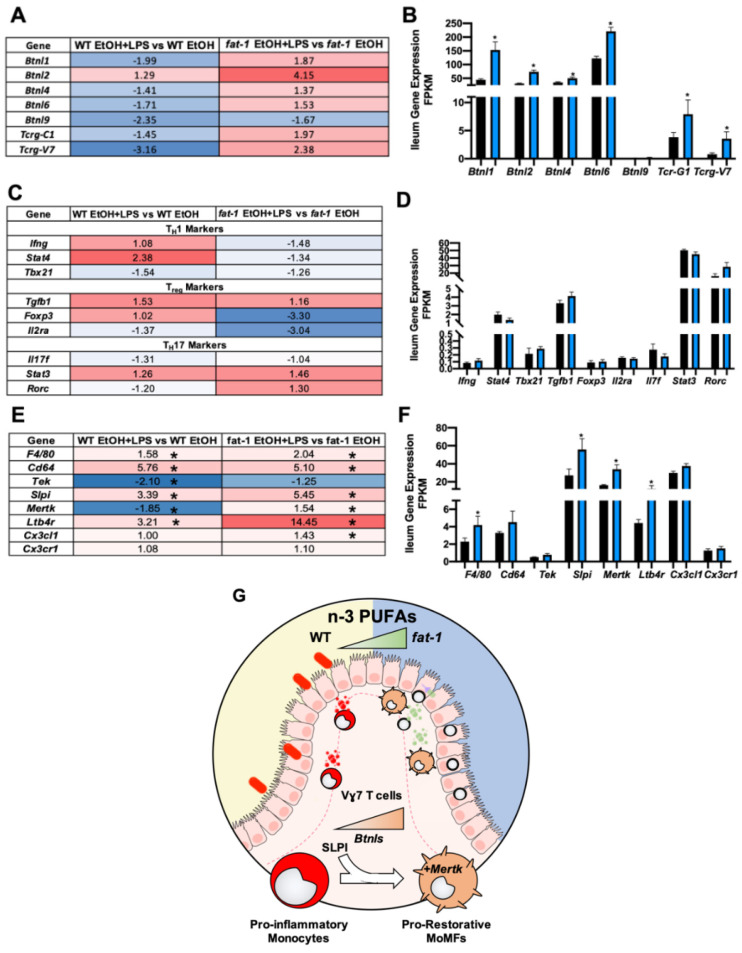
Increased n-3 PUFAs enhanced the ileum expression of Btnl-mediated T cell and pro-restorative macrophage gene signatures. (**A**) Heatmap fold change values for ileum *Btnl* and γδ T cell gene signatures for the WT EtOH + LPS vs. WT EtOH comparison and the *fat-1* EtOH + LPS vs. *fat-1* EtOH comparison. (**B**) Ileum *Btnl* and γδ T cell gene signature expression for WT EtOH + LPS and *fat-1* EtOH + LPS-treated mice. (**C**) Heatmap fold-change values for T_h_1, T_reg_, and T_h_17 cell gene markers for the WT EtOH + LPS vs. WT EtOH comparison and the *fat-1* EtOH + LPS vs. *fat-1* EtOH comparison. (**D**) Gene expression of T_h_1, T_reg_, and T_h_17cell gene markers in WT EtOH + LPS vs. *fat-1* EtOH + LPS-treated mice. (**E**) Heatmap fold change values for ileum pro-restorative macrophage markers. (**F**) Pro-restorative macrophage gene expression for WT EtOH + LPS and *fat-1* EtOH + LPS-treated mice. (**G**) Graphical representation of BTNLs, γδ T cells, and pro-restorative macrophages being enhanced in the ileum by n-3 PUFA enrichment. Statistical significance (*p* < 0.05) is denoted by an *.

Butyrophilin-like (Btnl) genes have recently been demonstrated to be expressed by intestinal epithelial cells (IECs) where they act as tethers to enhance the γδ T cell population and heighten defense response and tissue homeostasis [17,18]. The expression of *Btnl1/2* and *Tcrg-C1* (a γδ T cell marker) was decreased by EtOH in both WT and *fat-1* mice (Figure S2A,B). Interestingly, all *Btnl* and γδ T cell signature genes (other than *Btnl2*) were decreased in EtOH + LPS vs. EtOH-treated WT mice. In contrast, the same genes (other than *Btnl9*) were increased by EtOH + LPS vs. EtOH in *fat-1* mice (Figure 6A). Notably, the *Btnl* genes (*Btnl1/2/4/6/9*), *Tcr-G1*, and *Tcrg-V7* were all elevated in *fat-1* EtOH + LPS mice as compared to WT EtOH + LPS mice (Figure 6B). T_h_1, T_reg_, and T_h_17 cell gene markers were evaluated, but there were no statistical differences noted for WT EtOH + LPS vs. WT EtOH, *fat-1* EtOH+LPS vs. *fat-1* EtOH (Figure 6C) or *fat-1* EtOH + LPS vs. WT EtOH + LPS mice (Figure 6D).

Pro-restorative macrophages contribute to favorable intestinal tissue homeostasis and host immune responses [19]. Pro-restorative macrophage gene signatures were evaluated in the ileum of EtOH and PF WT and *fat-1* mice. The expression of *Cx3cl1* and *Ltb4r1* was decreased by EtOH in both genotypes but *Slpi* was only decreased in WT mice (Figure S2C). The expression of pro-restorative macrophage gene signatures was elevated in the EtOH + LPS vs. EtOH group in both WT and *fat-1* mice (except for *Tek* and *Mertk*) (Figure 6E). The expression of these pro-restorative macrophage genes was also increased in *fat-1* EtOH + LPS vs. WT EtOH + LPS mice with *Slpi*, *Mertk*, and *Ltb4r1* being significant (Figure 6F). Taken together, these data demonstrate that enhanced n-3 PUFAs increased ileum *Btnls*, γδ T cells, and pro-restorative *MertK+* macrophages in EtOH + LPS treated mice (Figure 6G). 

#### 2.6.2. Increased n-3 PUFAs Enhanced APRIL Gene Expression and IgA + B-Cell Markers

**Figure 7:**
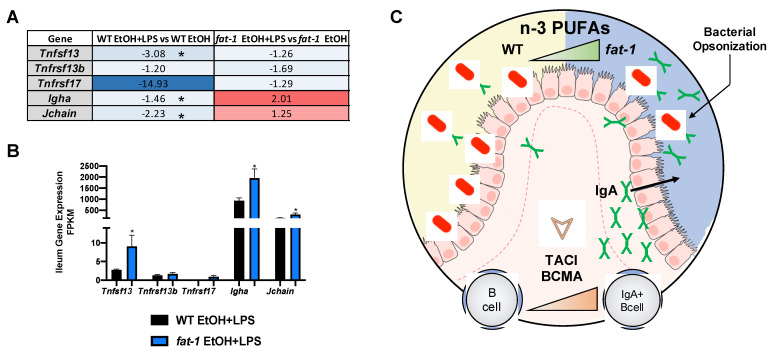
Increased n-3 PUFAs enhanced APRIL-signaling gene expression and IgA + B-Cell markers. (**A**) Heatmap fold-change values for APRIL signaling genes and IgA genes for the WT EtOH + LPS vs. WT EtOH comparison and the *fat-1* EtOH + LPS vs. *fat-1* EtOH comparison. (**B**) APRIL signaling and IgA gene expression for WT EtOH + LPS and *fat-1* EtOH + LPS-treated mice. (**C**) Graphical representation of enhanced APRIL signaling and IgA + B cells in the ileum of mice with n-3 PUFA enrichment. Statistical significance (*p* < 0.05) is denoted by an *.

Next, we evaluated APRIL signaling, an important pathway in intestinal B cell-mediated anti-bacterial response [20]. Activation of intestinal B cells by APRIL (*Tnfsf13*) signaling leads to the production of IgA which functions to mitigate bacterial expansion in the gut [21]. APRIL signals through its receptors TACI (*Tnfrsf13b*) and BCMA (*Tnfrs17*), which, in turn, switches B cells to IgA producing B cells [21]. The expression of *Tnfrsf17*, *Igha,* and *Jchain* was decreased by EtOH feeding in both genotypes while *Tnfsf13* was only decreased in WT EtOH mice (Figure S3). The expression of *Tnfrsf13b* was increased in *fat-1* EtOH vs. WT EtOH mice (Figure S3). *Tnfsf13, Tnfrsf13b, Tnfrsf17, Igha, a*nd *Jchain* gene expression was decreased by EtOH + LPS in both genotypes, except for *Igha* and *Jchain,* which were elevated in *fat-1* EtOH + LPS vs. *fat-1* EtOH mice (Figure 7A). When directly comparing the expression of these genes between *fat-1* EtOH + LPS vs. WT EtOH + LPS-treated mice, all were increased in *fat-1* mice (with *Tnfsf13*, *Igha*, and *Jchain* being significant) (Figure 7B). Collectively, these data suggest that enrichment of n-3 PUFAs increased IgA-producing B cells and APRIL signaling markers in the intestine of EtOH + LPS treated *fat-1* mice (Figure 7C).

#### 2.6.3. Increased n-3 PUFAs Attenuated the EtOH+LPS-Mediated Intestinal Pro-Fibrotic Gene Expression

**Figure 8:**
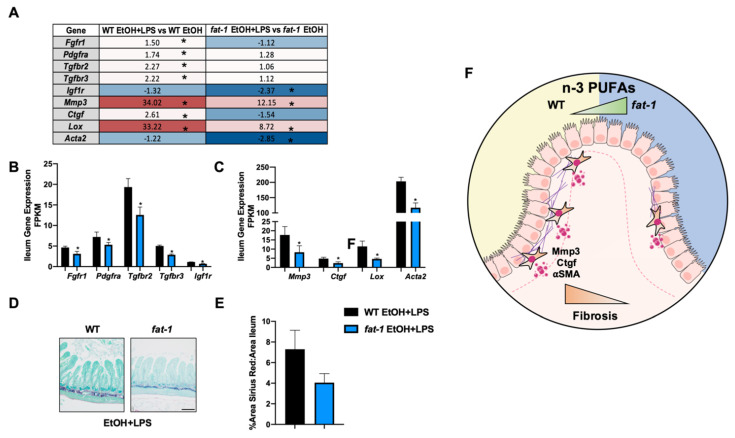
Increased n-3 PUFAs attenuated the EtOH + LPS mediated intestinal fibrosis. (**A**) Heatmap fold-change values for pro-fibrotic receptors and markers for the WT EtOH + LPS vs. WT EtOH comparison and the *fat-1* EtOH + LPS vs. *fat-1* EtOH comparison. (**B**) Gene expression of pro-fibrotic receptors in WT EtOH + LPS and *fat-1* EtOH + LPS-treated mice. (**C**) Gene expression of pro-fibrotic markers in WT EtOH + LPS and *fat-1* EtOH + LPS-treated mice. (**D**) Representative Images of Sirius red-stained ileal sections at 200× from WT EtOH + LPS and *fat-1* EtOH + LPS-treated (scale bar is 40 μm). (**E**) Quantification of Sirius red staining area relative to total ileum area for WT EtOH + LPS vs. *fat-1* EtOH + LPS mice. (**F**) Graphical representation of diminished intestinal fibrosis associated with enhanced n-3 PUFAs. Statistical significance (*p* < 0.05) is denoted by an *.

Chronic intestinal injury and inflammation may result in intestinal fibrosis [22]. Thus, we evaluated the expression of the pro-fibrotic receptors *Pdgfrα*, *Tgfbr2*, and *Igfr1*, which was increased by EtOH independent of genotype (Figure S4A), along with *Acta2,* an established marker of fibrosis (Figure S4B). Next, when comparing EtOH + LPS vs. EtOH in WT and *fat-1* mice, the expression of *Pdgfra*, *Tgfbr2*, *Tgfbr3*, *Mmp3*, and *Lox* was increased in both genotypes. The expression of *Igf1r* and *Acta2* was decreased by EtOH + LPS in both genotypes. *Fgfr1* and *Ctgf* were increased in WT mice but decreased in *fat-1* mice in response to EtOH + LPS vs. EtOH (Figure 8A). When directly comparing *fat-1* EtOH + LPS vs. WT EtOH + LPS mice, the expression of the pro-fibrotic receptors (*Fgfr1*, *Pdgfra*, *Tgfbr2*, *Tgfbr3*, *Igf1r*) was decreased in *fat-1* mice (Figure 8B). Pro-fibrotic gene signatures (*Mmp3*, *Ctgf*, *Lox*, *Acta2*) were downregulated in *fat-1* EtOH + LPS vs. WT EtOH + LPS-treated mice (Figure 8C). To histologically assess ileum fibrosis, Sirius red staining was performed, which revealed a lower extent of fibrotic scarring (although not significantly so) in *fat-1* EtOH + LPS relative to WT EtOH + LPS-treated mice (Figure 8D,E). In WT EtOH fed mice there was a significant increase in Sirius red staining relative to WT PF that was not observed in *fat-1* EtOH vs. *fat-1* PF mice (Figure S4C,D). Collectively, these data demonstrate that endogenous elevation of n-3 PUFAs diminished the expression of EtOH + LPS-induced intestinal fibrosis markers (Figure 8F).

## 3. Discussion

The detrimental effects of chronic alcohol exposure on intestinal tissue integrity and function are well known [11,23]. Specifically, excessive alcohol consumption is often associated with endotoxemia [24] as a result of increased gut permeability and leakage of gut-derived pathogens, including LPS, into circulation, affecting multiple tissues and organs including the gut. However, the underlying mechanisms of EtOH and EtOH + LPS-mediated gut alterations are not well understood. In this study, we aimed to identify global transcriptional changes in the intestine following chronic EtOH exposure and LPS-induced systemic inflammation. In order to examine the effects of n3 PUFAs in modulating the transcriptional responses, we utilized *fat-1* transgenic mice with endogenously elevated n-3 PUFA levels in all tissues. We observed that in response to EtOH + LPS vs. EtOH alone, WT mice experienced the greatest differential change in gene expression (2276 DEGs for WT vs. 1583 for *fat-1*). WT and *fat-1* mice had some similar transcriptional responses to EtOH + LPS vs. EtOH, including elevated *Lcn2* (the most induced gene in both genotypes), which is pro-inflammatory and recruits immune cells to sites of inflammation [25]. The expression of other inflammatory mediators was similarly increased in both genotypes, including *Tnf*, *Il1b*, and *Csf1* in the EtOH + LPS vs. EtOH group. *Csf1* is required to maintain the resident intestinal macrophage population [26], and these data suggest that in an EtOH-acute systemic inflammation model there is an increase in resident intestinal macrophages, independent of genotype. Further, the expression of smooth muscle contraction genes, including *Actg2*, was decreased in EtOH + LPS vs. EtOH mice in both genotypes as well. Loss of function mutations of *Actg2* lead to reduced intestinal peristalsis and correlate with disease severity in chronic intestinal pseudo-obstruction syndromes [27]. These data indicate that EtOH + LPS can reduce the expression of intestinal motility markers independent of n-3 PUFA enrichment; therefore, EtOH and LPS may promote a loss of intestinal peristalsis. LPS has been shown to disturb intestinal motility in a JNK dependent manner through upstream TNFα signaling [28]. The TNFα-JNK pathway may be less active in *fat-1* mice in response to EtOH + LPS, restoring intestinal motility, but future investigation is required to validate this concept.

WT and *fat-1* mice also had differential responses to EtOH + LPS vs. EtOH alone, including increased expression of chemokine and cytokine signaling genes in WT mice. Inflammation of the gut is common with chronic alcohol consumption, inflammatory bowel disease (IBD), and colitis. For example, *Cxcl1*, a chemokine that recruits neutrophils and is highly expressed in IBD [29], was highly expressed in WT but not *fat-1* mice in response to EtOH + LPS. Similarly, both IBD-associated pro-inflammatory mediators, *Il1a* and its receptor *Il1r* [30], were increased exclusively in WT EtOH + LPS-treated mice. These data indicate that n-3 PUFAs may function to dampen the intestinal immune response to EtOH and LPS. In contrast, the expression of lipid metabolism transcription factors such as *Nr1h3* and *Srebf1* were increased in only *fat-1* mice in response to EtOH+LPS vs. EtOH. Deficient intestinal lipid metabolism has previously been described in IBD [31]. Recently, the loss of *Nr1h3* was shown to exacerbate experimental colitis, and *NR1H3* expression was found to be reduced in IBD patients [32]. Therefore, endogenous enrichment of n-3 PUFAs may transcriptionally regulate lipid metabolism in the intestine via increased expression of *Nr1h3* which in turn elevates the expression of its target gene *Srebf1*.

There were also genesets decreased exclusively in the EtOH + LPS vs. EtOH groups in each genotype. The expression of genes involved in Wnt signaling were exclusively decreased in WT mice in response to EtOH + LPS, including *Axin2*, a transcriptional readout of Wnt signaling. Wnt signaling in intestinal stem cells is required for damage-induced regeneration of the intestinal epithelium (as in this EtOH-acute systemic inflammation model), but not for normal intestinal homeostasis [33]. Our data would suggest that n-3 PUFAs enhance the proliferative capacity of the intestinal epithelium during an EtOH-acute systemic inflammation model. Other ileum genes were exclusively decreased in *fat-1* EtOH + LPS vs. EtOH-treated mice. The expression of cytotoxic T cell markers, including *Thy1* and *Cd8b* was decreased exclusively in EtOH + LPS vs. EtOH *fat-1* mice. Cytotoxic T cells are elevated in IBD [34] and may exacerbate intestinal inflammation. Enrichment of n-3 PUFAs may function to reduce cytotoxic T cells stimulated by EtOH and LPS.

When directly comparing *fat-1* EtOH + LPS vs. WT EtOH + LPS-treated mice, we found an increase in the expression of xenobiotic metabolism genes in *fat-1* mice, including *Cyp2b10*, *Cyp3a11*, and *Nr1i3*. *Cyp2b10* and *Cyp3a11*, both of which are target genes for *Nr1i3*, are decreased in expression in ulcerative colitis [35]. Enrichment of n-3 PUFAs may increase the expression and transcriptional activity of *Nr1i3*, providing a robust xenobiotic metabolism response during EtOH and LPS-induced injury and inflammation. The expression of intestinal *Serpine1* was decreased in *fat-1* EtOH + LPS vs. WT EtOH + LPS mice as well. *SERPINE1* is elevated in IBD patients and links intestinal inflammation to coagulation, both of which are both factors involved in fibrosis [36]. Consumption of n-3 PUFAs in humans has been demonstrated to reduce *SERPINE1* in whole blood [37], but it may have similar effects on the intestine, as suggested by these data.

Previously we demonstrated that n-3 PUFA enrichment in *fat-1* mice reduced pro-inflammatory cytokine levels in the ileums of EtOH + LPS-exposed mice [15]. Here we report that the expression of pro-inflammatory markers was reduced in *fat-1* mice in contrast to pro-restorative immune cell markers, which were increased. Intraepithelial γδ T cells play a pro-restorative role in intestinal immune responses and tissue homeostasis. The expression of γδ T cells markers (*Tcrg-C1, Tcrg-V7*) was increased with elevated n-3 PUFAs after EtOH + LPS treatment. The effect of *fat-1* appears to be specific to ileum γδ T cells markers as other T cell population markers (T_h_1, T_h_17, T_reg_) were not altered. *Btnl* gene expression was also elevated by n-3 PUFA enrichment. BTNLs are expressed by IECs and enhance the recruitment of intraepithelial γδ T cells [17]. Intestinal mucosa γδ T cells regulate immunotolerance to pathogenic bacteria and promote intestinal wound healing [38]. Metabolites of n-3 PUFAs have been demonstrated to impact T cell functions but not γδ T cells specifically [39]. The increased expression of γδ T cell markers may be due to a n-3 PUFA effect on IEC *Btnl* gene expression. The transcriptional regulation of *Btnl* genes is understudied, but n-3 PUFAs may transcriptionally regulate *Btnl* gene expression through nuclear receptor activation [40]. IECs expressing more *Btnls* due to n-3 PUFA enrichment could explain the retention of γδ T cells. Enriched intestine γδ T cells may contribute to intestinal integrity and diminished inflammation. The expression of markers of monocyte-derived macrophages (MoMFs), which are pro-restorative immune cells, was increased in *fat-1* mice. MoMFs contribute to intestinal homeostasis and resolution of inflammation. Recently, a dysfunction in monocyte differentiation to MoMFs has been demonstrated in IBD [19]. Infiltrating monocytes can receive external signaling cues and differentiate to MoMFs at sites of inflammation; one such cue is SLPI. SLPI increases the number of MERTK+ pro-restorative macrophages [41]. The expression of both *Slpi* and *Mertk* was elevated in *fat-1* EtOH+LPS vs. WT EtOH+LPS-treated mice. It has been demonstrated that both *Mertk^-/-^* [42] and *Slpi^-/-^* [43] mice develop exacerbated intestinal inflammation. Our data suggest that n-3 PUFAs may enhance the pool of pro-restorative macrophages to help maintain tissue homeostasis and integrity.

Enrichment of n-3 PUFAs also affected other intestinal immune cell responses. Intestinal immune cells generate many antibodies, such as IgA, to mitigate bacterial overgrowth and maintain immune tolerance [44]. Secretory IgA is produced by B cells [45], which then undergoes transcytosis to reach the gut mucosal layer where it functions through immune exclusion to keep bacteria within the mucosa. Along with B cell-mediated prevention of bacterial translocation, the intestinal epithelial layer also maintains a physical barrier limiting gut permeability (e.g., ZO-1, and other tight junction proteins). Previously we found no prominent differences in intestinal tight junction genes or a reduction of plasma endotoxin in *fat-1* EtOH fed mice relative to WT EtOH fed mice [15]. In the current model mice were fed an EtOH containing diet and LPS was administered systemically, which can on its own disrupt gut barrier function [13]. In this study there were no differences in ileum tight junction genes (*Tjp1, Cldn, Ocln*) between *fat-1* EtOH + LPS vs. WT EtOH + LPS mice (data not shown). Here, we demonstrated that n-3 PUFA enrichment increased the expression of *Igha* and *Jchain* (the secretory IgA dimer linker) in the ileum. The expression of IgA + B cells is enhanced by APRIL (*Tnfsf13*) signaling [21]. APRIL is released by IECs following TLR activation (*e.g.*, LPS activation of TLR4), leading to B cell proliferation and IgA secretion [21]. In this study, we found that the expression of APRIL (*Tnfsf13*) was increased by n-3 PUFA enrichment, which may contribute to the observed effect on *Igha* expression. Previously, it has been shown that the dietary n-3 PUFAs eicosapentaenoic acid and docosahexaenoic acid can enhance cecal IgA levels [46], consistent with the findings of this study. It has also been demonstrated that chronic EtOH consumption reduces fecal levels of secretory IgA in mice [47]. In an alcohol-associated liver disease mouse model, *Igha^-/-^* mice did not develop exacerbated liver injury, which was partly explained by a compensatory upregulation of IgM [48], although intestinal pathology was not evaluated in this study. These data suggest that n-3 PUFAs enhanced expression of APRIL signaling components (ligand and receptors), which leads to enhanced IgA + B cell levels. This in turn would combat bacterial overgrowth and reduce bacterial-induced inflammation in the intestine.

In our study, fibrotic scarring of the ileum was increased by EtOH + LPS to a greater extent in WT vs. *fat-1* mice. Further, the expression of ileum pro-fibrotic receptors *Tgfbr2/3*, *Pdgfrα, Fgfr1* and *Igf1r* was decreased in *fat-1* EtOH + LPS vs. WT EtOH + LPS-treated mice along with *Ctgf, Mmp3, Lox,* and *Acta2*. LPS- and microbe-induced intestinal fibrosis has been demonstrated previously [14], and here we demonstrate that elevation of n-3 PUFAs can reduce this effect. Intestinal fibrosis occurs after repeated intestinal injury and inflammation leading to a buildup of scar tissue [22]. This in turn stiffens the intestinal tract reducing gut motility, while simultaneously exacerbating permeability and GI bleeds [22]. Myofibroblasts in the intestine proliferate during intestinal fibrosis due to pro-fibrotic growth factors such as CTGF [49]. Interestingly, *Ctgf* expression was reduced with elevated n-3 PUFAs which could contribute to the decreased expression of other myofibroblast markers (*Acta2*, *Lox*, and *Mmp3*). In hepatic stellate cells, n-3 PUFAs reduce *Ctgf* expression induced by EtOH [50]; here we show that this effect may also be present in intestinal stellate cells. Taken together, these data demonstrate that n-3 PUFAs are anti-fibrotic in the intestine during EtOH + LPS-mediated intestinal inflammation.

## 4. Materials and Methods

### 4.1. Experimental Study Design

Animal studies were approved by and performed in accordance with the guidelines of the University of Louisville Institutional Animal Care and Use Committee (IACUC). The IACUC protocol numbered 15423 was approved on 12 January 2016 by the IACUC ethics committee. Mice were housed in a temperature-controlled room with a 12 h light-dark cycle in a pathogen-free animal facility accredited by the Association for Assessment and Accreditation of Laboratory Animal Care. The *fat-1* transgenic mouse line [5] was used to investigate the effect of n-3 PUFA enrichment on EtOH and LPS-induced intestinal pathology. These mice express the *C. elegans fat-1* n-3 fatty acid desaturase in all tissues and therefore have increased n-3 PUFAs in the absence of dietary modification [15]. 8–10-week-old *fat-1* and WT male littermates were placed on a control (maltose dextrin) or EtOH-containing Lieber-DeCarli liquid diet (catalog numbers F1259SP and F1258SP, respectively. BioServ, Flemington, NJ, USA). Mice were fed for 6 weeks with an initial stepwise increase in EtOH concentration (0%, 1%, and 2% for two days each, 4% and 5% for one week each and then 6% for 3 weeks). EtOH-fed WT and *fat-1* mice were subjected to a one-time i.p. injection of LPS (5 mg/kg) 24 h before sacrifice to induce systemic inflammation (Figure 1A). RNASeq analysis of ileum tissue from WT PF (*n* = 4), *fat-1* PF (*n* = 4), WT EtOH fed (*n* = 3), *fat-1* EtOH fed (*n* = 5), WT EtOH + LPS (*n* = 4), and *fat-1* EtOH + LPS (*n* = 4) experimental mice was conducted and analyzed in this study.

### 4.2. Ileum Tissue Sample Acquisition and Histological Analysis

Mice were anesthetized with ketamine/xylazine (100/16 mg/kg) and ileum tissue was collected and either snap-frozen in liquid nitrogen and stored at −80 °C for further RNA isolation, or immediately fixed in 10% neutral buffered formalin and embedded in paraffin for Picrosirius red staining as a measure for intestinal fibrosis according to a standard protocol. Ileum sections were sectioned at 5 μm. Images were captured at 200× via an Olympus BX43 microscope and CellSens Software package (Olympus America, Breinigsville, PA, USA). Quantitation of ileum fibrosis was assessed by two independent investigators quantifying percent area of Sirius red staining relative to percent area of ileum tissue section in Image J as has been previously described [51]. 5 randomized images per ileum section were used for the Image J analysis.

### 4.3. Ileum Tissue RNA Isolation and Quality Analysis

Total ileum RNA was isolated using Trizol reagent (Thermo Fisher, Waltham, MA, USA) from murine ileal segments. This was followed by removal of any contaminating genomic DNA with DNase I (TURBO DNA-free kit, Thermo Fisher). RNA was further purified using the GeneJET RNA cleanup and concentration micro kit (Thermo Fisher). RNA integrity was determined by analysis on the Agilent Bioanalyzer 2100 (Agilent, Santa Clara, CA, USA) and only RNA samples with integrity values ranging from 7 to 9 were used for RNA-seq analysis.

### 4.4. Intestinal Tissue RNA-Sequencing

RNA sequencing was performed by the University of Louisville Center for Genetics in Molecular Medicine core facility using the TruSeq Stranded mRNA library preparation kit (part no. 20020594). The full RNASeq methodology has been reported previously [15].

### 4.5. RNA-seq Bioinformatics, Statistical Analysis, and Data Visualization

RNA-seq analysis was conducted by the NIH-funded Kentucky Biomedical Research Infrastructure Network Bioinformatics Core. The full details for this method have been published previously [15], but pertinent details are described below. The sequences were directly aligned to the *Mus musculus* reference genome assembly (mm10) using TopHat2 (version 2.0.13) [52] guided by Ensembl build 82 mouse transcripts. DEGs between experimental groups were identified using the Tuxedo suite of programs including cuffdiff2 (version 2.2.1) [53,54]. A *p*-value cutoff ≤ 0.05, *q*-value cutoff ≤ 0.05 with a fold change (FC) ≥2 was used to determine differential expression. DEGs that met this threshold were used for further analysis. Visualization of gene clusters and gene interactions for DEGs was conducted in Cytoscape [55]. Heatmap gradient coloration denotes log_2_ (FC) for the respective comparison and node size depicts relative connectivity from the Search Tool for the Retrieval of Interacting Genes/Proteins (STRING) database [56]. Similarly, Gene Ontology: Biological Processes (GO:BPs) and Kyoto Encyclopedia of Genes and Genomes (KEGG) Pathways that included the DEGs were conducted in Cytoscape [55]. Cytoscape analysis identified enriched GO:BPs (representative of DEGs increased), diminished GO:BPs (representative of DEGs decreased) for their respective comparisons. The top ten enriched and diminished GO:BPs were reported along with their associated false-discovery rate (FDR < 0.05). Similarly, STRING Analysis and gene clustering were conducted in Cytoscape followed by KEGG Pathway enrichment (FDR < 0.05). Gene clusters and their associated KEGG Pathway were used for DEGs increased and decreased for the respective comparisons. The GEO accession number for the RNAseq data reported in this paper is GSE133253. 

## 5. Conclusions

The ileal transcriptional responses in an EtOH-acute systemic inflammation model were described in WT and *fat-1* mice. Many transcriptional responses were similar between WT and *fat-1* mice in response to EtOH + LPS vs. EtOH, but others were distinct. This study identified ileum APRIL signaling and IgA + B cell gene signatures as being positively upregulated by n-3 PUFA enrichment in this model. In addition, the expression of pro-restorative macrophage and γδ T cell genes was increased by n-3 PUFA enrichment in contrast to pro-fibrotic genes, which were decreased in *fat-1* mice. Reduced fibrosis was confirmed by Sirius red staining. Follow-up studies are required to further validate these findings and to determine the implications in alcohol and acute inflammation- mediated intestinal pathology in humans. These data demonstrate that n3-PUFAs can significantly modify the transcriptional response to EtOH + LPS, which may mitigate many of the deleterious effects on the gut and therefore may be a therapeutic strategy to treat intestinal damage.

## Data Availability

Data are available in the publicly accessible GEO Data repository. Data can be found under the following accession number [GSE133253].

## Supplementary Materials

The following are available online at https://www.mdpi.com/1422-0067/22/4/1582/s1.

## Abbreviations

PUFAs	Polyunsaturated fatty acids
LPS	Lipopolysaccharide
EtOH	Ethanol
APRIL	A proliferation inducing ligand
WT	Wild type
GI	Gastrointestinal
SIRS	Systemic inflammatory response syndrome
DEGs	Differentially expressed genes
GO	Gene Ontology
NAD	Nicotinamide adenine dinucleotide
ECM	Extracellular matrix
PPAR	Peroxisome proliferator-activated receptor
AA	Amino acid
TLR	Toll-like receptor
cAMP	Cyclic adenosine monophosphate
BTNL	Butyrophilin-like
IEC	Intestinal epithelial cells
PF	Pair-fed
IBD	Inflammatory bowel disease
MoMFs	Monocyte derived macrophages
STRING	Search Tool for the Retrieval of Interacting Genes/Proteins (STRING) database
GO:BPs	Gene Ontology:Biological Processes
KEGG	Kyoto Encyclopedia of Genes and Genomes
