# Supplementary material for: Ileum Gene Expression in Response to Acute Systemic Inflammation in Mice Chronically Fed Ethanol: Beneficial Effects of Elevated Tissue n-3 PUFAs
Source: Int J Mol Sci. 2021 Feb 4;22(4):1582. doi: 10.3390/ijms22041582 (PMC7914826; doi:10.3390/ijms22041582)
Supplement: Supplementary file 1 [file ijms-22-01582-s001.pdf]

Supplemental Materials

Ileum Gene Expression in Response to Acute Systemic Inflammation in Mice Chronically Fed Ethanol: Beneficial Effects of Elevated Tissue n-3 PUFAs

Josiah E. Hardesty [1,2,†], Jeffrey B. Warner [1,2,†], Ying L. Song [1], Eric C. Rouchka [3], Craig J. McClain [1,2,4,5,6], Dennis R. Warner [1] and Irina A. Kirpich [1,2,4,5,*]

1 Division of Gastroenterology, Hepatology, and Nutrition, Department of Medicine, University of Louisville, Louisville, KY, USA; josiah.hardesty@louisville.edu (J.E.H.); jeffrey.warner.1@louisville.edu (J.B.W.); ying.song@louisville.edu (Y.L.S.); craig.mcclain@louisville.edu (C.J.M.); dennis.warner@louisville.edu (D.R.W.)
2 Department of Pharmacology and Toxicology, University of Louisville School of Medicine, Louisville, KY, USA
3 Department of Computer Science and Engineering, Speed School of Engineering, University of Louisville, Louisville, KY, USA; eric.rouchka@louisville.edu
4 University of Louisville Alcohol Center, University of Louisville School of Medicine, Louisville, KY, USA
5 University of Louisville Hepatology and Toxicology Center, University of Louisville School of Medicine, Louisville, KY, USA
6 Robley Rex Veterans Medical Center, Louisville, KY, USA
* Correspondence: i0kirp01@louisville.edu
† These authors contributed equally to this work.

Table S1. Top ten DEGs in WT EtOH+LPS vs WT EtOH.

Gene title	Fold change	*p*-value	*q*-value	Molecular Function
Lcn2	566.8	0.00005	0.00033973	Protease Binding
Fabp1	166.6	0.00005	0.00033973	Fatty Acid Binding
Saa3	140.1	0.00005	0.00033973	Chemoattractant
G6pc	88.3	0.00005	0.00033973	Glucose-6-Phosphatase
S100a8	80.8	0.00155	0.00676799	Protein Binding
Serpina3n	54.6	0.00005	0.00033973	Peptidase Inhibitor
Igkv3-1	47.5	0.00025	0.00143149	Antigen Binding
Ptx3	44.6	0.00005	0.00033973	Protein Binding
Fga	43.7	0.00115	0.00528216	Extracellular Matrix
Cxcl1	43.0	0.00005	0.00033973	Chemokine
Car1	-215	0.01065	0.0335852	Aryl esterase
Gsdmc4	-155.7	0.00005	0.00033973	Phosphatidylserine Binding
Gsdmc2	-149.9	0.00005	0.00033973	Phosphatidylserine Binding
Cyp2c55	-141.2	0.00005	0.00033973	Monooxygenase
Pla2g4c	-84.4	0.00005	0.00033973	Phospholipase
Gm8979	-71.8	0.00005	0.00033973	Unknown
9130208D14Rik	-57.4	0.00005	0.00033973	Unknown
Gm6086	-43.4	0.00005	0.00033973	Sulfotransferase
Slc37a2	-38.7	0.00005	0.00033973	Antiporter
Mme	-33.1	0.00005	0.00033973	Endopeptidase

Table S2. Top ten DEGs in *fat-1* EtOH+LPS vs *fat-1* EtOH.

Gene title	Fold change	*p*-value	*q*-value	Molecular Function
Lcn2	496.8	5.00E-05	0.00093986	Protease Binding
Lct	402.0	0.00005	0.00093986	Lactase
Gata4	134	0.0019	0.0193444	Transcription Factor
Gimd1	123.9	0.0027	0.0252945	Nucleotide Binding
Saa3	122.2	0.00005	0.00093986	Chemoattractant
Plb1	117.5	0.00005	0.00093986	Phospholipase
Ugt2a3	91.6	0.00005	0.00093986	Transferase
Ifit3	87.4	0.00005	0.00093986	RNA Binding
Apoa4	68.1	0.00005	0.00093986	Lipid Transport
Isg15	67.5	0.00005	0.00093986	Integrin Binding
Cyp2c55	-197.9	0.00005	0.00093986	Monooxygenase
Hao2	-143.9	0.00005	0.00093986	Oxidoreductase
Cyp2d34	-127.1	0.00005	0.00093986	Monooxygenase
Gsdmc2	-122.3	0.00005	0.00093986	Phosphatidylserine Binding
Gsdmc4	-99.1	0.00005	0.00093986	Phosphatidylserine Binding
Nov	-86.8	0.00005	0.00093986	Notch Binding
Cbs	-84.8	0.00005	0.00093986	Cysteine Synthase
Slc6a14	-48.2	0.00005	0.00093986	Symporter
Fa2h	-46.2	0.00005	0.00093986	Oxidoreductase

Table S3. Top ten DEGs shared between WT and *fat-1* mice in response to EtOH+LPS vs EtOH.

	***fat-1* EtOH+LPS vs *fat-1* EtOH**			**WT EtOH+LPS vs WT EtOH**			
**Gene title**	**fold**	***p*-value**	***q*-value**	**fold**	***p*-value**	***q*-value**	**Molecular Function**
Lcn2	496.8	0.00005	0.00093986	566.803824	0.00005	0.00033973	Protease Binding
Gata4	134	0.0019	0.0193444	12.6818599	0.00005	0.00033973	Transcription Factor
Gimd1	123.9	0.0027	0.0252945	6.40208746	0.00005	0.00033973	Nucleotide Binding
Saa3	122.2	0.00005	0.00093986	140.081757	0.00005	0.00033973	Chemoattractant
Plb1	117.5	0.00005	0.00093986	11.0081047	0.00005	0.00033973	Phospholipase
Ifit3	87.4	0.00005	0.00093986	41.6191885	0.00005	0.00033973	RNA Binding
Apoa4	68.1	0.00005	0.00093986	14.5408208	0.00005	0.00033973	Lipid Transport
Isg15	67.5	0.00005	0.00093986	23.8113806	0.00005	0.00033973	Integrin Binding
Usp18	67.2	0.00005	0.00093986	36.6686476	0.00005	0.00033973	Peptidase
Mx2	64.9	0.00005	0.00093986	28.7745743	0.00005	0.00033973	Nucleotide Binding
Car1	-5168.5	0.00035	0.00503675	-215	0.01065	0.0335852	Aryl esterase
Cyp2c55	-197.9	0.00005	0.00093986	-141.2	0.00005	0.00033973	Monooxygenase
Hao2	-143.9	0.00005	0.00093986	-27	0.00005	0.00033973	Oxidoreductase
Cyp2d34	-127.1	0.00005	0.00093986	-8.8	0.00005	0.00033973	Monooxygenase
Gsdmc2	-122.3	0.00005	0.00093986	-149.9	0.00005	0.00033973	Phosphatidylserine Binding
Gsdmc4	-99.1	0.00005	0.00093986	-155.7	0.00005	0.00033973	Phosphatidylserine Binding
Cbs	-84.8	0.00005	0.00093986	-16.9	0.00005	0.00033973	Cysteine Synthase
Slc6a14	-48.2	0.00005	0.00093986	-10.6	0.00005	0.00033973	Symporter
Fa2h	-46.2	0.00005	0.00093986	-2.7	0.00005	0.00033973	Oxidoreductase
St6galnac6	-30.7	0.00005	0.00093986	-4.4	0.00005	0.00033973	Sialyltransferase

Table S4. Top ten DEGs exclusive to WT EtOH+LPS vs WT EtOH mice.

Gene title	Fold change	*p*-value	*q*-value	Molecular Function
Fabp1	166.6	0.00005	0.00033973	Fatty Acid Binding
S100a8	80.7	0.00155	0.00676799	Protein Binding
Igkv3-1	47.5	0.00025	0.00143149	Antigen Binding
Ptx3	44.6	0.00005	0.00033973	Protein Binding
Fga	43.7	0.00115	0.00528216	Extracellular Matrix
Cxcl1	43	0.00005	0.00033973	Chemoattractant
Timp1	37.9	0.01665	0.0484773	Protease Inhibitor
Muc1	23.2	0.00645	0.0222319	Mucin
Adamts4	22.1	0.00935	0.0302396	Metallopeptidase
Marco	20.4	0.01405	0.0423881	Scavenger Receptor
9130208D14Rik	-57.4	0.00005	0.00033973	Unknown
Mme	-33.1	0.00005	0.00033973	Endopeptidase
Igkv2-109	-31.2	0.00005	0.00033973	Antigen Binding
Igkv4-53	-24.5	0.00005	0.00033973	Antigen Binding
Ighv14-4	-19.8	0.00005	0.00033973	Antigen Binding
Rn7sk	-17.8	0.0011	0.00508653	
Igkv6-13	-16.1	0.00005	0.00033973	Antigen Binding
Ighv1-52	-14.9	0.00005	0.00033973	Antigen Binding
Abca12	-14.8	0.00005	0.00033973	Antigen Binding
Igkv5-43	-11.6	0.00005	0.00033973	Antigen Binding

Table S5. Top ten DEGs exclusive to *fat-1* EtOH+LPS vs *fat-1* EtOH mice.

Gene title	Fold change	*p*-value	*q*-value	Molecular Function
Lct	402	0.00005	0.00093986	Lactase
Ugt2a3	91.6	0.00005	0.00093986	Transferase
Cyp2b10	64.6	0.00005	0.00093986	Monooxygenase
Enpp3	53	0.00005	0.00093986	Nucleic Acid Binding
Fpr1	33.8	0.0056	0.044064	G-protein Coupled Receptor
Igkv2-116	29.1	0.00315	0.0285008	Antigen Binding
Nr1i3	27.4	0.00025	0.00380465	Nuclear Receptor
S100a9	24.3	0.00465	0.0382219	Protein Binding
Slc23a1	23.4	0.0003	0.00442667	Transporter
Chil1	18.5	0.00005	0.00093986	Chitin Binding
Nov	-86.8	0.00005	0.00093986	Notch Binding
Grin3a	-45	0.00005	0.00093986	Ion Channel
Ighg2c	-32.8	0.0026	0.0246593	Antigen Binding
Npy4r	-29.2	0.0031	0.0281711	Peptide Hormone Binding
Gm11346	-28.9	0.0035	0.030899	Unknown
Gm2115	-25.5	0.0037	0.0321765	Unknown
Adamts18	-25.2	0.00465	0.0382219	Metalloendopeptidase
Nxpe2	-21.4	0.00005	0.00093986	Protein Binding
Syt2	-20.7	0.0015	0.0160349	SNARE Binding
Cyp2d10	-19.6	0.00005	0.00093986	Monooxygenase

Table S6. Top ten DEGs in *fat-1* EtOH+LPS vs WT EtOH+LPS mice.

Gene title	Fold change	*p*-value	*q*-value	Molecular Function
Lct	202.9	0.00005	0.00132348	Lactase
Cyp2b10	100.9	0.00005	0.00132348	Monooxygenase
Slc28a1	96.7	0.0002	0.00451313	Symporter
Mme	46.4	0.00005	0.00132348	Endopeptidase
Gm766	45.9	0.00005	0.00132348	Unknown
Enpp3	45.2	0.00005	0.00132348	Nucleic Acid Binding
Cyp3a11	45.1	0.00005	0.00132348	Monooxygenase
Ugt2a3	39	0.00005	0.00132348	Transferase
Asah2	30.5	0.00005	0.00132348	Sphingolipid Metabolism
Slc25a45	25.2	0.00005	0.00132348	ATP Transporter
Zfp865	-18.6	0.00005	0.00132348	Nuclei Acid Binding
Igkv4-69	-14.6	0.0023	0.0311507	Antigen Binding
Hal	-8	0.0002	0.00451313	Ammonia Lyase
Ighg1	-7.1	0.00005	0.00132348	Antigen Binding
Gm20703	-6.7	0.003	0.0378	Unknown
Fa2h	-6.3	0.00005	0.00132348	Oxidoreductase
Gm21498	-6.1	0.0004	0.007956	Unknown
Gm11821	-6	0.0039	0.04563	Unknown
Gm42974	-5.2	0.00075	0.0127345	Unknown
B230208H11Rik	-5.1	0.0009	0.0146902	Unknown

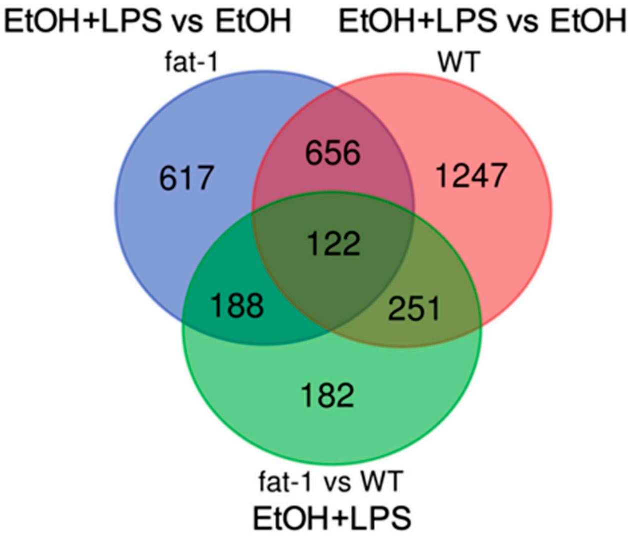

Figure S1. Venn diagram illustrating the number of significant ileum genes shared between statistical comparisons. The blue circle encompasses the number of significant ileum genes for the *fat-1* EtOH+LPS vs *fat-1* EtOH comparison. The red circle encompasses the number of significant ileum genes from the WT EtOH+LPS vs WT EtOH comparison. The green circle encompasses the number of significant ileum genes from the *fat-1* EtOH+LPS vs WT EtOH+LPS comparison.

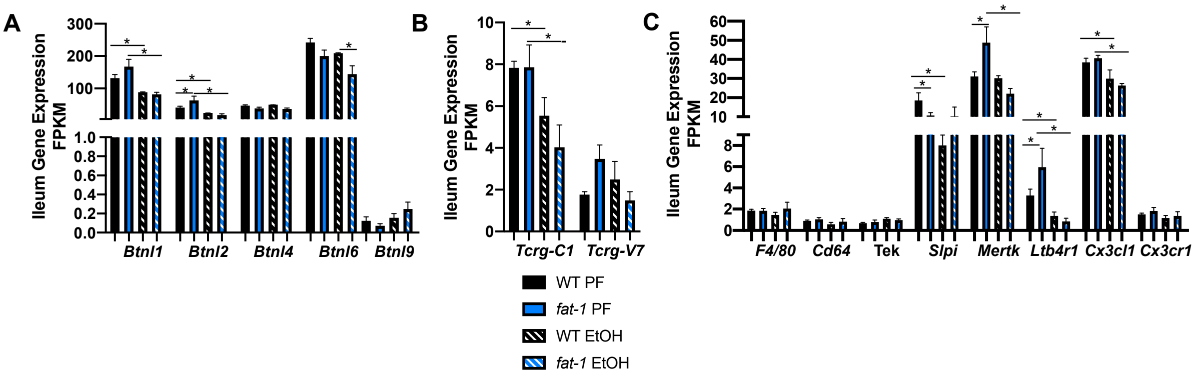

Figure S2. Ileum expression of BTNL-mediated T cell and pro-restorative macrophage gene signatures after EtOH feeding in WT and fat-1 mice. (**A**) Ileum Btnl and (**B**) γδ T cell gene signature expression for WT PF, fat-1 PF, WT EtOH, and fat-1 EtOH mice. (**C**) Pro-restorative macrophage gene expression for WT PF, fat-1 PF, WT EtOH, and fat-1 EtOH mice.

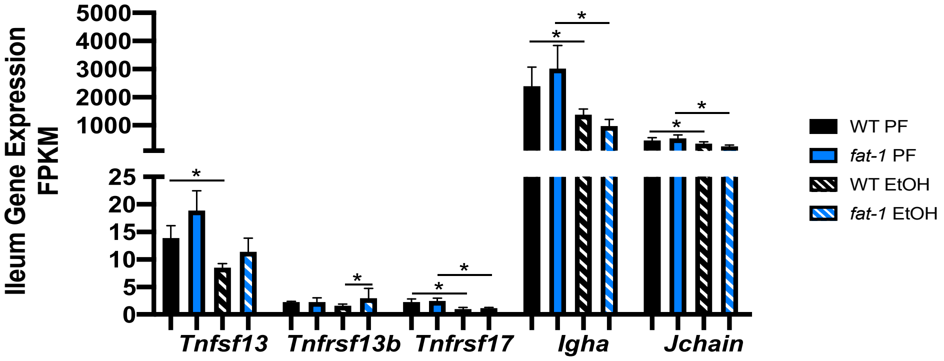

Figure S3. Ileum expression of APRIL-signaling genes and IgA+ B-Cell gene markers. APRIL signaling and IgA gene expression for WT PF, fat-1 PF, WT EtOH, and fat-1 EtOH mice.

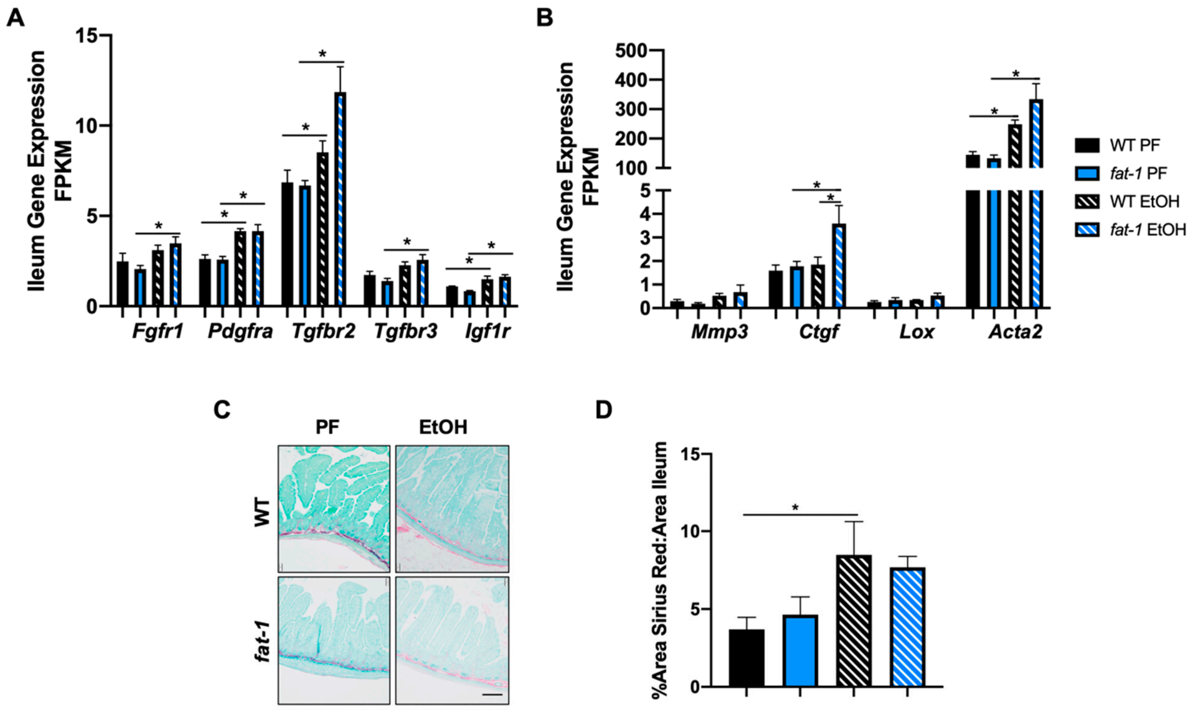

Figure S4. Ileum expression of fibrosis markers and histological ileum fibrosis. (**A**) Ileum gene expression of pro-fibrotic receptors in WT PF, fat-1 PF, WT EtOH, and fat-1 EtOH mice. (**B**) Ileum gene expression of pro-fibrotic markers in WT PF, fat-1 PF, WT EtOH, and fat-1 EtOH mice. (**C**) Representative images of Sirius red-stained ileal sections at 200X from WT PF, WT EtOH, *fat-1* PF, and *fat-1* EtOH mice (scale bar is 40 µm. (**D**) Quantification of are of sirius red staining relative to total ileum area.
